# Supplementary material for: Factors of Anti-Vascular Endothelial Growth Factor Therapy Withdrawal in Patients with Neovascular Age-Related Macular Degeneration: Implications for Improving Patient Adherence
Source: J Clin Med. 2021 Jul 14;10(14):3106. doi: 10.3390/jcm10143106 (PMC8306388; doi:10.3390/jcm10143106)
Supplement: Supplementary file 1 [file jcm-10-03106-s001.zip › jcm-1239452-supplementary.pdf]

Supplementary materials

Supplemental Methods

All data, including diagnoses and treatments, were self-reported and could not be verified by cross-checks with medical records. In terms of the anticipated sample size for this survey, we estimated that 180–240 patients would complete this exploratory survey for Cohort 1 and 100 for Cohort 2. No power analysis or sample size calculation was done.

The main questionnaire (**Supplemental Tables 2 and 3**) was assessed and validated internally via face-to-face interviews with six nAMD patients individually (four who had discontinued anti-VEGF therapy and two continuing anti-VEGF therapy). Following these interviews, the pre-defined reasons were modified in terms of the clarity of the wording, avoiding duplicated meanings, and inclusion of the most expected reasons.-

All respondents were also asked to complete validated Japanese versions of the EQ-5D-5L to assess health-related quality of life and the PAM-13 to measure patient activation levels [1]. The PAM-13 is a 13-item questionnaire designed to assess the patient's knowledge, skill, and confidence with self-managing their health or disease [2-4]. Each item comprises four possible responses (disagree strongly/disagree/agree/agree strongly/not applicable), which are converted to a scale of 0–100, where higher levels indicate more proactive attitudes towards managing own health.

References

1. Herdman, M.; Gudex, C.; Lloyd, A.; Janssen, M.; Kind, P.; Parkin, D.; Bonsel, G.; Badia, X. Development and preliminary testing of the new five-level version of EQ-5D (EQ-5D-5L). *Qual Life Res* **2011**, *20*, 1727-1736, doi:10.1007/s11136-011-9903-x.
2. Fujita, E.; Kuno, E.; Kato, D.; Kokochi, M.; Uehara, K. Development and validation of the Japanese version of the Patient Activation Measure 13 for Mental Health. *Seishin Igaku* **2010**, *52*, 765-772 (in Japanese).
3. Hibbard, J.H.; Mahoney, E.R.; Stockard, J.; Tusler, M. Development and testing of a short form of the patient activation measure. *Health Serv Res* **2005**, *40*, 1918-1930, doi:10.1111/j.1475-6773.2005.00438.x.
4. Hibbard, J.H.; Stockard, J.; Mahoney, E.R.; Tusler, M. Development of the Patient Activation Measure (PAM): conceptualizing and measuring activation in patients and consumers. *Health Serv Res* **2004**, *39*, 1005-1026, doi:10.1111/j.1475-6773.2004.00269.x.

Table S1. Patient questionnaire.

Questions	Responses
**Screening questions**	
Have you been diagnosed with age-related macular degeneration (AMD) by a physician?	• Yes<br>• No<br>• Not sure
Have you received anti-VEGF therapy to treat your AMD?	• Yes<br>• No<br>• Not sure
Has it been 6 months or longer since you last received anti-VEGF therapy?	• Yes (Cohort 1)<br>• No (Cohort 2)
If Cohort 1:<br>Was it your doctor's decision to not have anti-VEGF therapy in the last few months?	• Yes (Cohort 1-1)<br>• No (Cohort 1-2)
If Cohort 1-1:<br>How would you describe your current situation regarding anti-VEGF therapy?	• I completed treatment<br>• I am still continuing follow-up visits
Sex	• Male<br>• Female
Age	• <50 years<br>• 50–59 years<br>• 60–69 years<br>• 70–79 years<br>• ≥80 years
Place of residence	• Japan scale
**General questions**	
Are you living with family?	• Yes<br>• No
Who are they?	• Spouse/partner<br>• Children<br>• Other
Are you currently employed?	• Yes<br>• No
What is your highest level of education?	• Middle school<br>• High school<br>• Vocational school<br>• Junior college<br>• University<br>• Post-graduate school
How often do you smoke?	• Everyday<br>• Sometimes<br>• Used to smoke<br>• Never smoked
**Comorbidities**	
Other than AMD, what illnesses do you have? (multiple choice)	• Glaucoma<br>• Cataract<br>• Cerebrovascular disease<br>• Heart disease<br>• Diabetes mellitus<br>• Hypertension<br>• Hyperlipidemia<br>• Cancer<br>• Other<br>• None
**Eye-related questions**	

Regarding your eye condition, what do you find difficult in daily life? (multiple choice)	• To recognize people’s faces or objects<br>• To read books or newspapers<br>• To drive<br>• To go out alone<br>• Other<br>• No particular difficulties
What is your current visual acuity?	• 0.01–0.1<br>• 0.2–0.3<br>• 0.4–0.6<br>• 0.7–1.0<br>• ≥1.2<br>• Not sure
**Diagnosis-related questions**	
When were you diagnosed with AMD?	• Within 1 year<br>• Within 3 years<br>• Within 5 years<br>• Within 10 years<br>• ≥10 years
Do you have AMD in one eye or both eyes?	• One eye<br>• Both eyes
What was your level of understanding of AMD at the beginning of your diagnosis/treatment?	• Good<br>• Quite good<br>• Not quite good<br>• Not good at all
**Anti-VEGF therapy related questions**	
Type of hospital	• Eye clinic (primary care)<br>• Eye hospital with referral<br>• Eye hospital without referral (personal choice of secondary care)
Time to get to hospital	• <30 minutes<br>• ≥30 minutes<br>• ≥1 hour<br>• ≥1.5 hours
How did (do) you travel to hospital	• By car<br>• By taxi<br>• By public transport (train, bus, etc.)<br>• By bicycle<br>• On foot
Did (does) someone accompany you to hospital?	• Yes<br>• No
How long have you continued anti-VEGF therapy? (approximate time from the first to last injection)	• <3 months<br>• ≥3 months<br>• ≥1 year<br>• ≥3 years<br>• ≥5 years
Frequency of visits	• Every month<br>• Every 2 months<br>• Every 3 months<br>• Every 6 months<br>• Neither applies
Frequency of injections	• Every month<br>• Every 2 months<br>• Every 3 months<br>• Every 6 months<br>• Neither applies

What was the cost per visit when you had (have) an injection?	• <10,000 JPY<br>• 10,000–29,999 JPY<br>• 30,000–49,999 JPY<br>• ≥50,000 JPY
What is your relationship with your doctor?	• Very good<br>• Good<br>• Neither good nor bad<br>• Not good<br>• Not good at all
What is your current level of understanding of AMD?	• Good<br>• Quite good<br>• Not quite good<br>• Not good at all

AMD, age-related macular degeneration; VEGF, vascular endothelial growth factor.

Table S2. Main questionnaire: reasons for discontinuing anti-VEGF therapy in Cohort 1.

**Topic**	***C1-1:*** **Other than the doctor's decision, please specify whether each of the statements below is true in explaining your reason for discontinuing anti-VEGF therapy.**<br>***C1-2:*** **Please specify whether each of the statements below is true in explaining your reason for discontinuing anti-VEGF therapy.**
Efficacy	• Because the treatment was ineffective<br>• Because there was no deterioration in vision
Finance	• Because of financial burden
Understanding	• Because I was not sure why I should continue the treatment
Visits	• Because I had to visit hospital too often<br>• Because transport to hospital was inconvenient<br>• Because treatment was time-consuming including long wait times<br>• *If you were in need of a companion*, because it was hard to find someone who could take me to hospital
Treatment	• Because treatment required injections too many times<br>• Because it was scary to have an injection in the eye<br>• Because the feeling of the injection was unpleasant<br>• Because I was dissatisfied with the doctor
Etc.	• Because I had another disease I had to prioritize over AMD<br>• Because I was concerned about side effects

All reasons were presented in a random order, and had four possible responses: definitely true, mostly true, mostly false, definitely false. AMD, age-related macular degeneration; VEGF, vascular endothelial growth factor.

Table S4. Reasons for treatment discontinuation in Cohort 1.

	**Cohort 1-1** (discontinued due to doctor's decision) (*n* = 185)					**Cohort 1-2** (discontinued due to their own decision) (*n* = 22)				
	**True** (= Definitely true + Mostly true)	Definitely true	Mostly true	Mostly false	Definitely false	**True** (= Definitely true + Mostly true)	Definitely true	Mostly true	Mostly false	Definitely false
There was no deterioration in vision	**65 (35.1)**	36 (19.5)	29 (15.7)	26 (14.1)	94 (50.8)	**15 (68.2)**	7 (31.8)	8 (36.4)	3 (13.6)	4 (18.2)
The treatment was ineffective	**36 (19.5)**	10 (5.4)	26 (14.1)	34 (18.4)	115 (62.2)	**10 (45.5)**	7 (31.8)	3 (13.6)	5 (22.7)	7 (31.8)
Financial burden	**24 (13.0)**	3 (1.6)	21 (11.4)	33 (17.8)	128 (69.2)	**12 (54.5)**	6 (27.3)	6 (27.3)	2 (9.1)	8 (36.4)
It was scary to have an injection in the eye	**21 (11.4)**	4 (2.2)	17 (9.2)	34 (18.4)	130 (70.3)	**8 (36.4)**	4 (18.2)	4 (18.2)	4 (18.2)	10 (45.5)
Treatment was time-consuming including long waiting times	**20 (10.8)**	4 (2.2)	16 (8.6)	33 (17.8)	132 (71.4)	**9 (40.9)**	2 (9.1)	7 (31.8)	7 (31.8)	6 (27.3)
The feeling of the injection was unpleasant	**18 (9.7)**	6 (3.2)	12 (6.5)	31 (16.8)	136 (73.5)	**5 (22.7)**	2 (9.1)	3 (13.6)	5 (22.7)	12 (54.5)
I was not sure why I should continue the treatment	**16 (8.6)**	5 (2.7)	11 (5.9)	33 (17.8)	136 (73.5)	**10 (45.5)**	7 (31.8)	3 (13.6)	5 (22.7)	7 (31.8)
I had to visit hospital too often	**12 (6.5)**	1 (0.5)	11 (5.9)	39 (21.1)	134 (72.4)	**4 (18.2)**	0	4 (18.2)	11 (50.0)	7 (31.8)
Treatment required injections too many times	**12 (6.5)**	0	12 (6.5)	38 (20.5)	135 (73.0)	**5 (22.7)**	0	5 (22.7)	4 (18.2)	13 (59.1)
I was dissatisfied with the doctor	**10 (5.4)**	2 (1.1)	8 (4.3)	29 (15.7)	146 (78.9)	**4 (18.2)**	2 (9.1)	2 (9.1)	9 (40.9)	9 (40.9)
Transport to hospital was inconvenient	**8 (4.3)**	0	8 (4.3)	28 (15.1)	149 (80.5)	**7 (31.8)**	1 (4.5)	6 (27.3)	6 (27.3)	9 (40.9)
I had another disease I had to prioritize over AMD	**8 (4.3)**	2 (1.1)	6 (3.2)	23 (12.4)	154 (83.2)	**3 (13.6)**	0	3 (13.6)	4 (18.2)	15 (68.2)
I was concerned with side effects	**7 (3.8)**	1 (0.5)	6 (3.2)	37 (20.0)	141 (76.2)	**3 (13.6)**	0	3 (13.6)	6 (27.3)	13 (59.1)
If you were in need of a companion, it was hard to find someone who could take me to hospital	**4 (2.2)**	1 (0.5)	3 (1.6)	24 (13.0)	157 (84.9)	**0**	0	0	7 (31.8)	15 (68.2)

Values are *n* (%). AMD, age-related macular degeneration.

Table S5. Reasons for dissatisfaction with treatment in Cohort 2 (n = 65).

	**True** (= Definitely true + Mostly true)	Definitely true	Mostly true	Mostly false	Definitely false
Treatment is time-consuming with long waiting times	**50 (76.9)**	20 (30.8)	30 (46.2)	8 (12.3)	7 (10.8)
Financial burden	**46 (70.8)**	22 (33.8)	24 (36.9)	9 (13.8)	10 (15.4)
Treatment is ineffective	**38 (58.5)**	9 (13.8)	29 (44.6)	20 (30.8)	7 (10.8)
Feeling of the injection is unpleasant	**34 (52.3)**	10 (15.4)	24 (36.9)	25 (38.5)	6 (9.2)
I am scared of having an injection in the eye	**33 (50.8)**	9 (13.8)	24 (36.9)	24 (36.9)	8 (12.3)
Injections are required too often	**31 (47.7)**	7 (10.8)	24 (36.9)	24 (36.9)	10 (15.4)
I have to visit the hospital too often	**27 (41.5)**	5 (7.7)	22 (33.8)	27 (41.5)	11 (16.9)
Transport to hospital is inconvenient	**22 (33.8)**	4 (6.2)	18 (27.7)	26 (40.0)	17 (26.2)
I am concerned about side effects	**20 (30.8)**	2 (3.1)	18 (27.7)	27 (41.5)	18 (27.7)
It is unclear why I should continue treatment	**19 (29.2)**	4 (6.2)	15 (23.1)	32 (49.2)	14 (21.5)
I have another disease more severe than AMD	**15 (23.1)**	5 (7.7)	10 (15.4)	22 (33.8)	28 (43.1)
I am dissatisfied with the doctor	**12 (18.5)**	0	12 (18.5)	29 (44.6)	24 (36.9)
If you are in need of a companion, it is hard to find someone who can take me to hospital	**9 (13.8)**	3 (4.6)	6 (9.2)	13 (20.0)	43 (66.2)

Values are n (%). AMD, age-related macular degeneration.
